# Analysis of Mortality Change Rate from Temperature in Summer by Age, Occupation, Household Type, and Chronic Diseases in 229 Korean Municipalities from 2007–2016

**DOI:** 10.3390/ijerph16091561

**Published:** 2019-05-04

**Authors:** Jongchul Park, Yeora Chae, Seo Hyung Choi

**Affiliations:** Korea Environment Institute, 370 Sicheong-daero, Sejong 30147, Korea; jcpark@kei.re.kr (J.P.); shchoi@kei.re.kr (S.H.C.)

**Keywords:** heat waves, mortality, age, occupation, household type, chronic diseases, temperature distribution, Korea

## Abstract

This study analyzed mortality change rate (MCR: daily change rate of mortality at a given temperature per average summer mortality) for 229 municipalities in Korea considering age, occupation, household type, chronic diseases, and regional temperature distribution. We found that the MCR for heat wave differs depending on socioeconomic factors and the temperature distribution in the region. The MCRs for the elderly (≥65 years of age), outdoor workers, one-person households, and chronic disease patients start to increase at lower temperatures and react more sensitively to temperature than others. For the socioeconomic factors considered in this study, occupation was found to be the most significant factor for the MCR differences (outdoor workers 1.17 and others 1.10 above 35 °C, *p* < 0.01). The MCRs of elderly outdoor workers increased consistently with temperature, while the MCRs of younger outdoor workers decreased at 33 °C, the heat wave warning level in Korea. The MCRs in lower temperature regions start to increase at 28 °C, whereas the MCRs start to increase at 30 °C in higher temperature regions. The results of this study suggest that heat wave policies should be based on contextualized impacts considering age, occupation, household type, chronic disease, and regional temperature distribution.

## 1. Introduction

Heat waves have become one of the most significant health concerns globally as well as in Korea. The increase in extreme temperature events is affecting a variety of conditions relevant to human health, such as ischemic stroke, ischemic heart disease, acute myocardial infarction, angina pectoris, heat-related illness, and mental illness all over the worldwide [1,2,3,4,5,6,7,8,9]. Severe heat waves in Korea have also significantly increased mortality and morbidity, especially in 1994 and 2018 [10,11]. Heat waves also have adverse physical impacts: heat-related diseases occur more frequently and the mortality rate increases during extreme heat episodes.

The effects of the global 2018 heat wave highlighted the importance and urgency of having sophisticated heat wave policies. The damage from the 2018 heat wave was reported from all over the world [3,10,12,13,14]. In Korea, 48 deaths from heat-related diseases were reported—a figure that was twice that from the last three years. In 2018, all records related to high temperatures, such as daily maximum temperature, daily minimum temperature, sunshine hours, heat wave days, and tropical nights, reached the highest ever documented since 1907. Korea has legislated heat waves as a form of natural disaster [15].

The impacts of heat waves are not only determined by their severity and frequency, but also by the socioeconomic factors, age, occupation, income, and gender of those it affects. Elderly people are more vulnerable to high temperature [16,17,18] than younger people, and people with low incomes are more vulnerable than people with high incomes [18,19,20,21]. This is because the elderly and people with low incomes are less able to protect themselves and to respond promptly [22]. Occupation is also an important factor that affects a heat wave’s impacts. Blue-collar workers (relative risk 1.06) are more vulnerable to high temperatures than white-collar workers (relative risk 1.01) [23]. 

The effects of temperature on health are also different depending on the climate characteristics of the affected region. Lowe et al. showed that northern Europe is more sensitive to heat than southern Europe. In Denmark, deaths from temperature start to occur at temperatures 5 °C lower than that at which deaths start to occur in southern Portugal [24]. Gómez-Martín et al. insisted that response action to avoid heat waves could be influenced by one’s own personal experiences or one’s community’s experiences with heat waves [25].

Many studies on temperature have revealed the statistical significance of increases in mortality and morbidity due to increasing temperatures (e.g., [18,20,26,27,28,29]. Most studies focus on relative impacts individually by age, occupation, income, and climate conditions [4,5,11,18,20,29]. The impacts of extreme high temperatures are contextualized in specific regions reflecting the multiple factors described above. However, previous studies have not provided enough information for establishing customized heat waves policies for vulnerable groups at the regional level. For this study, we attempted to quantify changes in mortality rate from temperature considering physical factors (temperature), socioeconomic factors (age, occupation, household type, chronic diseases), and temperature distribution in 229 regions in Korean in order to provide customized heat wave policies by region and by group.

## 2. Methods

This study analyzed the demographic characteristics of 229 basic units of local government in Korea and examined the impacts of mortality from temperature. The study used daily maximum temperature and daily mortality data from 2007 to 2016 (June to August). Mortality data was obtained from Microdata Integrated Service from Statistics Korea [30]. Meteorological data was obtained from the Korea Meteorological Administration’s meteorological data release portal [31] (Table 1).

Mortality data, including cause of death, age at the time of death, occupation, and marital status, were obtained from the Statistics Korea. Total mortality was considered to include all deaths except those from external causes (International Classification of Diseases 10th Revision (ICD-10) codes A to R); demographic characteristics included age, household type, occupation, and chronic diseases. People were divided in those 65 those years of age and older (the “elderly”) and those less than 65 years of age, and occupations were classified as outdoor and others. The former included agricultural, forestry, and fisheries workers; device and machine operators and assemblers; construction workers; and military personnel. Households were divided into one-person households and others. Chronic diseases included heart diseases (ICD-10 I2–I5), liver diseases (ICD-10 K70–K77), diabetes mellitus (ICD-10 E10–E14), and hypertension-related diseases (I10–I15) [16,32,33,34,35].

A total of 535,495 people were considered in the study, which covered over a ten-year period; 402,769 of the people included in the study were over 65 years of age. Those from one-person households and outdoor workers accounted for 276,824 and 90,266 deaths, respectively. The factors considered for this time were age, outdoor workers, one-person households, and chronic diseases (Table 2).

Meteorological data were obtained from 440 automated weather systems operated by the Korea Meteorological Administration and interpolated into 1 km regular grids and zonal statistics for each region. The Gaussian process regression model was used for the former and only the temperatures of urban and agricultural regions were extracted using zonal statistics.

The mortality change rate (MCR), which is the daily change rate of mortality at a given temperature per average summer mortality, was analyzed after it was calculated using the following Equation (1):

MCR = ln(Mt + 1) / log(Ms + 1)(1)

where Mt is the average daily mortality rate above a specific temperature and Ms is the average daily mortality rate during the summer.

**Figure 1:**
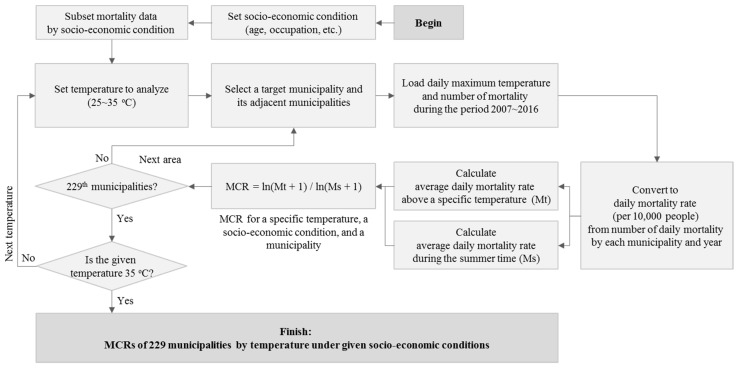
Flowchart of mortality change rate (MCR) calculation by temperature under given socio-economic conditions.

MCR is calculated by the following procedure (Figure 1). Socio-economic conditions to be analyzed are set and mortality data corresponding to socio-economic conditions are distinguished from the data set. Next, the temperature to analyze is set. The specific temperature was applied at intervals of 1 °C from 25 °C to 35 °C. Then, the target municipality and its adjacent areas is selected with temperature and mortality data obtained from 2007 to 2016. The data of adjacent municipalities are considered by using a spatial smoothing method because the confidence interval of the analysis results are widened when only a single sample from the region is used. Daily mortality was converted to daily mortality rate in order to consider the changes in total population. Daily mortality data is used to calculate Ms and Mt. Daily mortality data above a specific temperature is used to calculate Mt for the 10-year period. MCR is calculated by taking the natural logarithm to Ms and Mt. Logarithmic transformation is used to consider offset regional differences in population size, and 1 is added to Ms and Mt to remove errors when the mortality rate is 0. MCR is iteratively calculated by municipalities and 1 °C.

**Figure 2:**
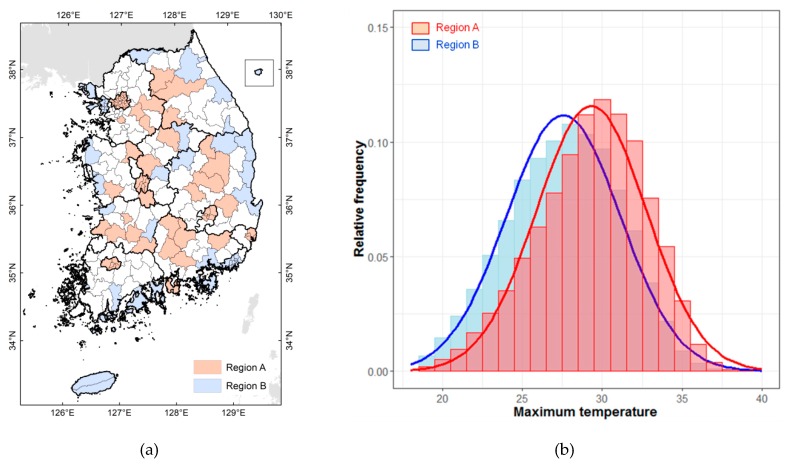
Temperature distribution in Korea, by region. (**a**) Regional distribution of average maximum daily summer temperature; (**b**) Distribution of average daily maximum summer temperature by region.

The study area was ranked based on the average daily maximum temperature in the summer over the last 20 years (1997–2016) and classified into A regions (areas with higher temperatures) and B regions (areas with lower temperatures). In Korea, maximum temperatures in summer are high in inland metropolitan and basin areas but low in areas influenced by the ocean and in rural areas, including high mountainous areas. Figure 2 shows two types of regions categorized based on the average maximum summer temperature. A regions have relatively high temperatures and B regions have relatively low temperatures (Figure 2a). There is an average difference of 2 °C in the maximum daily temperatures of the two types of region; the distribution of temperatures in the histogram for A regions is shifted to the right because high temperatures are frequent (Figure 2b).

## 3. Results

### 3.1. Impacts of Temperature in South Korea

The Korea Meteorological Administration heat wave warning system issues level-1 and level-2 heat wave warnings. Level-1 is issued when the daily maximum temperature of 33 °C or higher is expected to last for more than two days, and level-2 is issued when the daily maximum temperature of 35 °C or higher is expected to last for more than two days.

**Figure 3:**
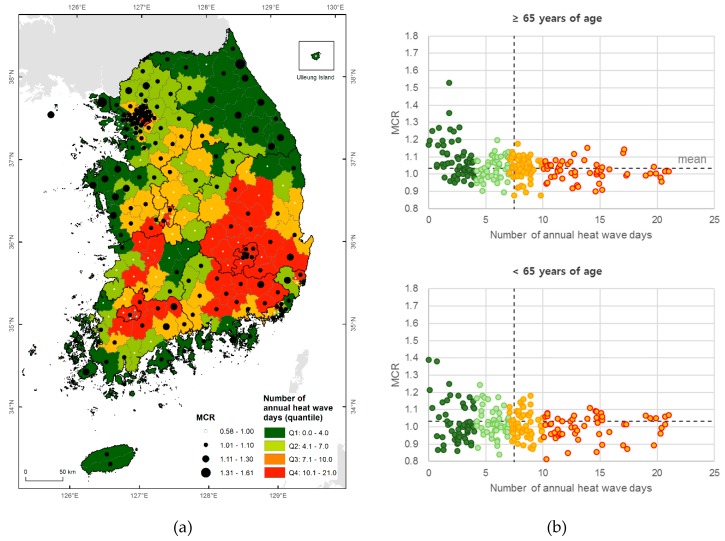
Number of heat wave days (33 °C or higher) and MCR of 33 °C per region in Korea for June–August 2007–2016. (**a**) Spatial distribution of heat wave days; (**b**) Scatter plot by ages.

The health impacts from temperature are not caused only by temperature. The MCR of 33 °C and number of heat wave days (33 °C or higher) are not significantly correlated; the northeastern region of Korea has fewer heat wave days, while its MCR is relatively high (Figure 3a). This discrepancy in spatial distribution can be seen in people of all ages (Figure 3b). This shows that it is difficult to explain the health effects of temperature using only temperature characteristics, and shows that local characteristics such as demographics, socioeconomic conditions, and other factors should also be considered.

**Figure 4:**
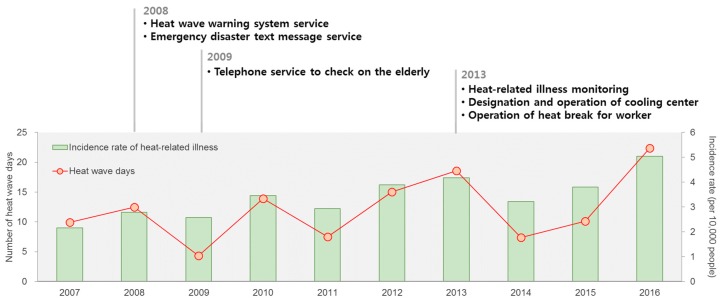
Number of heat wave days (33 °C or higher) and heat-related illness (HRI) incidence rates in South Korea since 2007.

Figure 4 is the number of heat wave days (33 °C or higher) and the heat-related illness (HRI) incidence rates in South Korea since 2007. The incidence rate is the number of outpatients per 10,000 population. It was calculated using HRI outpatient records that are obtained from the National Health Insurance Service. The eligibility criterion of the study population is all outpatients with a primary diagnosis of ICD-10 T67 and no visit within 7 days. This suggests that current policies have limitations in terms of managing the health impacts of heat waves. Despite multiple heat wave policies, including an emergency text service, the operation of cooling centers, and a telephone service for vulnerable groups, the HRI incidence rate has not improved.

### 3.2. Mortality Change Rate (MCR) by Age, Occupation, Household Type, and Chronic Disease

**Figure 5:**
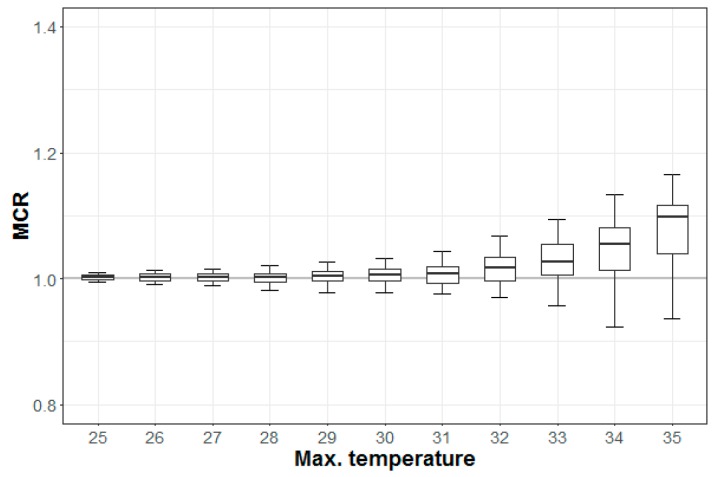
Mortality change rate (MCR) for total death by maximum temperature (10/90 percentile ranges).

The MCR increased with temperature in most regions during the period 2007–2016 (Figure 5). The MCRs in 75% of all regions reached 1.0 above 33 °C. This study focused on these regions to determine the key factors of the impacts of temperature. 

**Figure 6:**
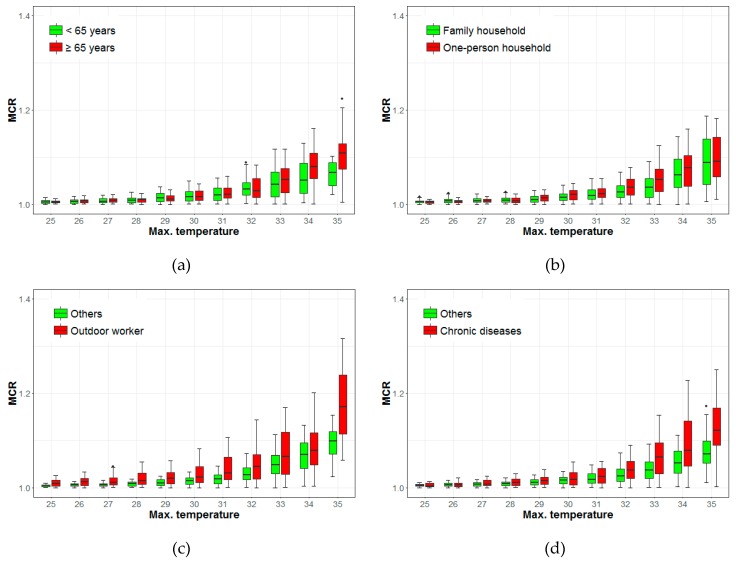
Mortality change rates (MCRs) for maximum temperatures, as organized by age, household type, occupation, and chronic diseases. (**a**) Age; (**b**) Household type; (**c**) Occupation; (**d**) Chronic Diseases.

The degree to which the MCR increases with temperature varies with different socioeconomic conditions. There is a statistically significant difference (0.028, 95% CI: 0.12–0.038, *p* < 0.001) between the MCRs for the elderly and those below the age of 65 when the maximum daily temperature is 34 °C. The median MCRs for the elderly and those under 65 years of age are 1.11 and 1.07, respectively, at a maximum daytime temperature of 35 °C (Figure 6a). The MCRs for one-person and family households showed differences when temperatures ranged from 30 °C to 33 °C. The differences are 0.006 (95% CI: 0.001–0.007, *p* < 0.01) and 0.016 (95% CI: 0.010–0.023, *p* < 0.001) at 30 °C and 33 °C, respectively. The median MCR of a one-person household is slightly higher than of a family household when the temperature ranges from 32–34 °C; however, the MCRs of the two groups are similar at 35 °C (Figure 6b). The MCR for outdoor workers showed a marked difference from the MCRs for all other classifications at all temperatures: the median MCR value for the former is 1.17 and that for the latter is 1.10 at 35 °C (Figure 6c). The difference is 0.073 (95% CI: 0.037–0.127, *p* < 0.01) at 35 °C. There was a statistically significant difference between those with chronic diseases and others at temperatures above 28 °C. The minimum difference was 0.003 (95% CI: 0.001–0.005, *p* < 0.01) at 28 °C and the maximum difference was 0.050 (95% CI: 0.012–0.086, *p* < 0.05) at 35 °C. The MCRs of those with chronic diseases was 1.12 at 35 °C; for others, that value was 1.07 (Figure 6d). Among all factors, the differences in the MCRs were most obvious among the occupations. More information about the difference of MCR medians and 95% confidence intervals between two groups can be found in in the Appendix A (Table A1).

**Figure 7:**
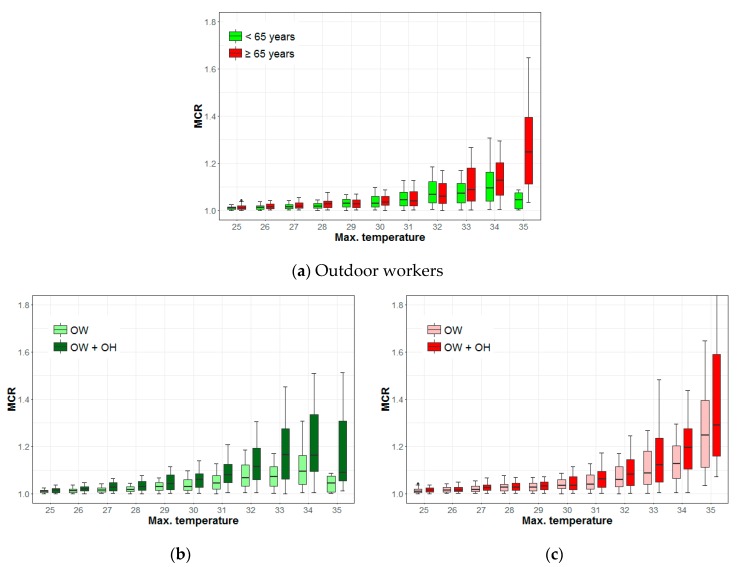
Mortality change rates (MCRs) of outdoor workers (OW) based on age, occupation, and household type (OH: one-person household). (**a**) Outdoor workers; (**b**) <65 years; (**c**) ≥65 years (elderly).

Figure 7 shows the differences in the MCRs of outdoor workers given age and a combination of other factors; the result shows a distinct difference at 33 °C (0.014, 95% CI: 0.015–0.056, *p* < 0.01). The median MCR for the elderly is 1.25, while that for those under 65 years old is 1.04 at 35 °C (Figure 7a). The MCR of one-person household outdoor workers under 65 years old is higher than that for those under 65 years of age. There was a statistically significant difference in the MCRs of the two groups. The MCR of outdoor workers under 65 years of age decreases above the temperature of 33 °C and drops sharply at 35 °C (Figure 7b). There was a significant difference in the MCRs of elderly one-person household outdoor workers compared to elderly family household outdoor workers above the temperature of 31 °C. The differences are 0.020 (95% CI: 0.003–0.027, *p* < 0.05) at 31 °C and 0.069 (95% CI: 0.023–0.089, *p* < 0.01) at 34 °C, respectively. The MCR of elderly outdoor workers increases steadily with rising temperatures regardless of the combination of other factors (Figure 7c). More information about the difference of MCR medians and 95% confidence intervals between two groups can be found in the Appendix A (Table A2).

Temperatures of 33 °C and 35 °C correspond to warning level-1 and warning level-2, respectively, for Korea’s heat wave warning system. There was a significant difference in the MCR patterns according to age, which suggests that there may be a clear difference in risk perception and behavioral patterns for heat waves for the two age groups. The difference in the MCRs is evident from the combination of factors, which indicates that socioeconomic factors have an important effect on the health of young people.

### 3.3. Risks of Mortality to Outdoor Workers Based on Climate

**Figure 8:**
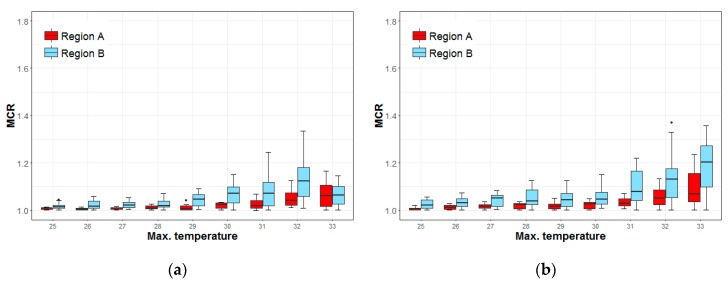
Mortality change rates (MCRs) of outdoor workers in different regions, by age, at maximum temperatures. (**a**) <65 years; (**b**) ≥65 years.

Figure 8 shows the MCRs of elderly outdoor workers and those under 65 years old in A regions and B regions. In A regions, the MCR of the younger workers increases gradually with the rising temperatures. In B regions, the MCR increases until a temperature of 32 °C but decreases sharply at 33 °C. The MCR of B regions was higher than that of A regions below 32 °C, but there was no difference between the regions at 33 °C (Figure 8a). The MCR of older workers increases gradually with temperature in both A and B regions. The MCR of the B regions is higher than that of the A regions and the increase in the slope is also steeper. The maximum difference is 0.134 (95% CI: 0.51–0.17, *p* < 0.001) at 33 °C. The increase in the MCR in the A regions is 0.04 (1.03 to 1.07) and the increase in the B region was 0.15 (1.05 to 1.20) (Figure 8b).

**Figure 9:**
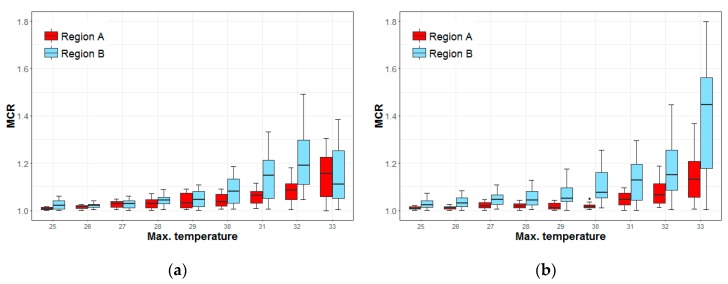
Mortality change rates (MCRs) of outdoor workers from one-person households, in different regions, by age, and at maximum temperatures. (**a**) <65 years; (**b**) ≥65 years.

The MCR patterns for one-person household outdoor workers are similar to those of all outdoor workers. There were statistically significant differences between the two types of regions at temperatures from 30 °C to 32 °C for the MCR of those under 65 years of age but no statistical difference at 33 °C. The differences are 0.045 (95% CI: 0.016–0.072, *p* < 0.01) at 30 °C and 0.104 (95% CI: 0.081–0.209, *p* < 0.001) at 32 °C (Figure 9a). The MCR for the elderly showed statistically significant differences at all temperatures (Figure 9b). More information about the difference of MCR medians and 95% confidence intervals between two groups can be found in the Appendix A (Table A3).

These results indicate that outdoor workers in areas with relatively low temperatures are susceptible to high temperature regardless of their age. The continuous increase in the MCRs of the elderly in both A and B regions after 33 °C (the heat wave warning level-1) indicates that the current heat wave warning system and its policies are not sufficient for this group.

The rapid decline observed in the MCR of those under 65 years at 33 °C in B regions suggests that the heat wave warnings and policies may have caused them to change their behavior. In addition, the high MCR in the region before temperatures reach 33 °C implies that young people may be working at a high intensity in relatively hot conditions (although not at the heat wave warning level) when they do not have previous experience of dealing with high temperatures.

## 4. Discussion

This study analyzed the MCRs of outdoor workers by age, household type, pre-existing chronic health condition (a chronic disease), and the distribution of temperatures in their region. Outdoor workers were most sensitive to temperature increases. Table 3 summarizes the MCRs of outdoor workers. 

The impacts of temperature increase when the proportion of time an individual spends working outside is high and when physical exertion levels while working are high [36,37]. Outdoor workers who experienced heat-related illness are highly correlated with exposure to extreme high temperature (95.7%) and work after struggling to sleep in tropical nights (78.7%) [38]. Outdoor workers who are vulnerable to heat waves are 224 difficult to manage their working conditions voluntarily Outdoor workers who are vulnerable to heat waves are difficult to manage their working conditions voluntarily. It is difficult to manage the working conditions of outdoor workers who are vulnerable to heat waves if the implementation of changes to working conditions are made solely on a voluntary basis. Policy management for these workers needs to be enhanced.

The sensitivity of outdoor workers to temperature was higher in one-person households. The influence was relatively higher in young people (aged less than 65 years). In addition, household type and pre-existing chronic disease were important factors not just for the elderly but younger people as well. This indicates that further attention should be given to people living alone and with chronic disease.

Social isolation is a critical risk factor of heat-related mortality [39]. People who live alone, live on the top floor, and stay mainly in the house showed a high odds ratio for heat-related deaths during the Chicago heat wave of 1995 [40]. Impacts of heat waves are disproportionately felt by elderly one-person households who have low incomes, weak social networks, and suffer from serious illnesses during the events [41]. Recently, it was stated that policies to prevent social isolation, such as community-based active monitoring programs, would reduce the impacts from heat waves [42]. However, more studies on social isolation factor in heat wave research are needed. This study showed that social isolation is a critical factor to determine heat wave mortality.

The MCR of young outdoor workers starts to decrease when heat wave warnings are given (Table 3). However, the MCR of elderly outdoor workers increased consistently with temperature regardless of age, household type, and regional temperature distributions. This implies that heat wave policies may not have been effective for this group. Elderly outdoor workers mainly work in the fields of agriculture, forestry, and fishing. It is difficult to follow guidelines and policies. Heat wave policies are shared via media, short message service, and apps in Korea, making it difficult for the elderly to obtain heat wave information via electronic devices. Young people are prone to accommodate experience, information, education, and policies about heat waves, all of which is useful for reducing their heat impacts [25]. If elderly people also receive the appropriate information at the individual level, the heat-evasive behavior increases [43]. This study indicated that information about the impacts of heat waves should be more actively transmitted to elderly people.

We found that mortality would increase at lower temperatures in the lower temperature regions (B regions). In lower temperature regions, the MCRs of both the elderly and young people showed a steep increase above 31 °C. This means that the heat wave warning system, which is based on daily maximum temperatures (level-1: 33 °C, level-2: 35 °C) may be limited in terms of its ability to reduce the health impacts in those regions. Health impacts vary with different climate zones, as has been shown in studies in Europe [24], the U.S. [44], and China [29]. We found that health impacts vary in the meso-climate zone in Korea. Thus, heat wave policies need to be customized to the temperature distribution of each region, as well as socio-economic factors like age, occupation, and household type.

This study did not consider lag effects. We focused on impacts from temperature distribution and socio-economic conditions. Although previous studies showed the impact of the lag effect varies with region, climate factors, and exposure [44,45], lag effects of heat waves occur in a short period of time, often within a few days [5,7,16,26,46,47,48,49].

The duration and severity of heat waves are projected to increase in the 21st century [50]. The aging and polarization trends occurring in Korea are likely to increase the health impacts from future heat waves [26]. Korea has become an “aged society” because of its low fertility rate and an increasing population of the aged; the proportion of the elderly in the population is expected to reach 25.0% in 2030 and 43.9% in 2060 [51]. According to Kim et al. mortality from heat waves is expected to increase five times in the 2060s under the representative concentration pathway 4.5 scenario [18]. It is important to understand the differences in the impacts of heat waves according to socioeconomic characteristics. Carefully designed policies based on contextualized impacts at the local level are required to prevent further heat wave damages in the future.

## 5. Conclusions

This study analyzed MCRs in 229 municipalities in Korea while considering age, occupation, household type, chronic diseases, and regional temperature distribution. We found that the MCRs for one-person households of the elderly, outdoor workers, and people with chronic diseases are relatively higher compared to other groups. Of these, a significant difference was observed between the MCRs of outdoor workers and others. 

The patterns of the MCR with temperature varies with the age of the people affected. The MCR of elderly outdoor workers increased steadily with temperature, but that of young outdoor workers decreased after the heat wave warning level, especially in B regions. These results suggest that young outdoor workers in these regions are not responding adequately to high temperatures before the heat wave warnings are issued. This also means that current heat wave warnings and policies may not be effective for elderly outdoor workers. It implies a need for contextualized heat wave policies considering key factors. This study found that regional temperature distribution is one of the key factors that should be considered to determine heat wave impacts. It is suggested that it is necessary to consider regional temperature distributions when setting heat wave warning levels and building practical and effective policies.

## Figures and Tables

**Table 1:** Data used to study mortality from temperature.

Data	Classify and Attribute		Source
Mortality (2007–2016)	Total	All deaths except from external causes	https://mdis.kostat.go.kr
	Ages	65 years old or more (elderly)	
		Under 65 years old	
	Household	One-person household	
		Family household	
	Job	Outdoor worker	
		Other	
	Diseases	Chronic diseases	
		Other	
Weather and Climate	Daily maximum temperature from AWS ^1^ (2007–2016)		https://data.kma.go.kr
	Daily maximum temperature from ASOS ^2^ (1997–2016)		

^1^ Automated Weather System, ^2^ Automated Synoptic Observation System.

**Table 2:** Deaths in the population of Korea during the summer from 2007 to 2016.

Classes	Factors	Population
Total	Total deaths (excluding external causes)	535,495
Age	65 years old or more (elderly)	402,769
	Under 65 years old	132,726
Household	One-person	276,824
	Family	258,671
Job	Outdoor	90,266
	Others	445,229
Diseases	Chronic diseases	106,468
	Others	507,621
Combinations	≥65 years + Outdoor	64,808
	<65 years + Outdoor	25,458
	≥65 years + Outdoor + One-person	23,628
	<65 years + Outdoor + One-person	9191

**Table 3:** The MCR median values of outdoor workers by age, household type, and regional temperature.

Factors	30 °C	33 °C Warning Level-1	35 °C Warning Level-2
Total deaths	1.01	1.04	1.10
Others	1.01	1.05	1.10
Outdoor workers	1.02	1.05	1.17
65 years old or more	1.03	1.09	1.25
One-person household	1.04	1.12	1.29
A Regions (areas with higher temperatures)	1.03	1.07	
B Regions (areas with lower temperatures)	1.05	1.20	
Under 65 years old	1.03	1.07	1.04
One-person household	1.06	1.16	1.09
A Regions (areas with higher temperatures)	1.02	1.06	
B Regions (areas with lower temperatures)	1.07	1.06	

## Appendix A

**Table A1:** The difference in median values of MCR by age, household type, occupation, and chronic diseases along with 95% confidence interval.

Max.T.	Age (≥65 Years vs. <65 Years)	Household Type (One-Person vs. Family)	Occupation (Outdoor vs. Others)	Chronic Diseases (Chronic Diseases vs. Others)
25		−0.001 (−0.002–0.000) **	0.004 (0.004–0.007) ***	
26		−0.001 (−0.002–0.000) *	0.006 (0.004–0.009) ***	
27	0.002 (0.000–0.003) .	−0.001 (−0.003–0.000) .	0.005 (0.005–0.010) ***	0.000 (0.000–0.004) *
28			0.007 (0.007–0.014) ***	0.003 (0.001–0.005) **
29	−0.002 (−0.005–0.000) .		0.009 (0.007–0.014) ***	0.004 (0.002–0.006) **
30		0.006 (0.001–0.007) **	0.008 (0.009–0.019) ***	0.001 (0.001–0.009) **
31			0.013 (0.015–0.028) ***	0.006 (0.002–0.010) **
32		0.009 (0.005–0.014) ***	0.017 (0.012–0.028) ***	0.012 (0.005–0.017) ***
33		0.016 (0.010–0.023) ***	0.018 (0.011–0.034) ***	0.028 (0.019–0.037) ***
34	0.028 (0.012–0.038) ***		0.008 (0.003–0.031) *	0.026 (0.026–0.055) ***
35	0.042 (0.022–0.072) **		0.073 (0.037–0.127) **	0.050 (0.012–0.086) *

Confidence level: . 90%, * 95%, ** 99%, *** 99.9%; Max.T.: maximum temperature.

**Table A2:** The differences in median values of MCR by age, household type, and occupation along with 95% confidence intervals for outdoor workers.

Max.T.	≥65 Years vs. <65 Years	One-Person and <65 Years vs. <65 Years	One-Person and ≥65 Years vs. ≥65 Years
25		0.003 (0.001–0.007) **	0.004 (0.000–0.006) *
26	0.003 (0.000–0.007) .	0.008 (0.003–0.011) ***	
27	0.003 (0.002–0.010) **	0.014 (0.008–0.017) ***	0.008 (0.002–0.011) **
28	0.009 (0.005–0.015) ***	0.014 (0.009–0.020) ***	
29		0.013 (0.009–0.026) ***	
30		0.029 (0.012–0.034) ***	
31		0.035 (0.026–0.052) ***	0.020 (0.003–0.027) *
32		0.046 (0.033–0.073) ***	0.022 (0.003–0.037) *
33	0.014 (0.015–0.056) **	0.092 (0.071–0.130) ***	0.035 (0.014–0.078) **
34	0.031 (−0.004–0.057) .	0.067 (0.058–0.142) ***	0.069 (0.023–0.089) **
35	0.203 (0.127–0.316) ***	0.045 (0.017–0.260) *	0.042 (−0.005–0.451) .

Confidence level: . 90%, * 95%, ** 99%, *** 99.9%; Max.T.: maximum temperature.

**Table A3:** The differences in median values of outdoor workers’ MCR between two temperature regions and accompanying 95% confidence intervals.

Max.T.	<65 Years	≥65 Years	<65 Years and One-Person	≥65 Years and One-Person
25	0.005 (0.004–0.015) **	0.016 (0.012–0.025) ***	0.014 (0.007–0.027) **	0.014 (0.010–0.027) ***
26	0.011 (0.007–0.029) **	0.020 (0.013–0.030) ***	0.005 (0.000–0.012) .	0.020 (0.014–0.034) ***
27	0.013 (0.007–0.024) **	0.035 (0.016–0.038) ***		0.029 (0.012–0.042) **
28	0.009 (0.004–0.024) **	0.017 (0.020–0.048) ***	0.014 (0.001–0.027) *	0.024 (0.015–0.049) **
29	0.040 (0.018–0.048) ***	0.028 (0.017–0.045) ***		0.040 (0.023–0.064) ***
30	0.046 (0.029–0.066) ***	0.019 (0.022–0.055) ***	0.045 (0.016–0.072) **	0.059 (0.056–0.124) ***
31	0.052 (0.023–0.074) **	0.050 (0.037–0.088) ***	0.086 (0.046–0.128) ***	0.084 (0.036–0.121) **
32	0.082 (0.043–0.115) ***	0.081 (0.043–0.123) ***	0.104 (0.081–0.209) ***	0.084 (0.040–0.154) **
33		0.134 (0.051–0.147) ***		0.316 (0.148–0.345) ***

Confidence level: . 90%, * 95%, ** 99%, *** 99.9%; Max.T.: maximum temperature.
